# Age-Dependent Differences in Radiation-Induced DNA Damage Responses in Intestinal Stem Cells

**DOI:** 10.3390/ijms251810213

**Published:** 2024-09-23

**Authors:** Guanyu Zhou, Tsutomu Shimura, Taiki Yoneima, Akiko Nagamachi, Akinori Kanai, Kazutaka Doi, Megumi Sasatani

**Affiliations:** 1 Department of Experimental Oncology, Research Institute for Radiation Biology and Medicine, Hiroshima University, Hiroshima 754-8553, Japan; d223678@hiroshima-u.ac.jp; 2 Department of Environmental Health, National Institute of Public Health, Saitama 351-0197, Japan; 3 School of Medicine, Hiroshima University, Hiroshima 754-8551, Japan; 4 Department of Molecular Oncology, Research Institute for Radiation Biology and Medicine, Hiroshima University, Hiroshima 754-8553, Japan; 5 Department of Computational Biology and Medical Sciences, Graduate School of Frontier Sciences, The University of Tokyo, Chiba 277-8561, Japan; 6 Department of Radiation Regulatory Science Research, Institute for Radiological Sciences, National Institutes for Quantum Science and Technology, Chiba 263-8555, Japan

**Keywords:** DNA damage response, radiation, age at exposure, cell cycle, DNA repair, stem cell, intestinal crypts, apoptosis, p53 activation, gene expression variability, Lgr5+ intestinal stem cells

## Abstract

Age at exposure is a critical modifier of the risk of radiation-induced cancer. However, the effects of age on radiation-induced carcinogenesis remain poorly understood. In this study, we focused on tissue stem cells using *Lgr5-eGFP-ires-Cre^ERT2^* mice to compare radiation-induced DNA damage responses between Lgr5+ and Lgr5- intestinal stem cells. Three-dimensional immunostaining analyses demonstrated that radiation induced apoptosis and the mitotic index more efficiently in adult Lgr5- stem cells than in adult Lgr5+ stem cells but not in infants, regardless of Lgr5 expression. Supporting this evidence, rapid and transient p53 activation occurred after irradiation in adult intestinal crypts but not in infants. RNA sequencing revealed greater variability in gene expression in adult Lgr5+ stem cells than in infant Lgr5+ stem cells after irradiation. Notably, the cell cycle and DNA repair pathways were more enriched in adult stem cells than in infant stem cells after irradiation. Our findings suggest that radiation-induced DNA damage responses in mouse intestinal crypts differ between infants and adults, potentially contributing to the age-dependent susceptibility to radiation carcinogenesis.

## 1. Introduction

The evaluation of cancer risk from radiation exposure has become a significant concern for the safe use of radiation, especially given the increasing opportunities for radiation exposure owing to advancements in medical care, industrial applications, and nuclear power plants. Age at exposure is a factor that modifies the risk of radiation-induced carcinogenesis, with the risk of radiation-induced cancer generally higher in childhood than in adulthood. This trend has been observed in studies on Hiroshima and Nagasaki A-bomb survivors, where an increased risk of overall solid cancer incidence and mortality was noted among those exposed at younger ages [1,2,3]. However, the impact of age at exposure on radiation-related cancer risk varies according to tissue type. For instance, the excess relative risk per Gy for lung cancer increases with increasing age at exposure, whereas that for breast cancer is highest around menarche [4]. Colorectal cancer is the third most prevalent cancer worldwide, comprising approximately 10% of all diagnosed cancer cases [5]. It is also the second-leading cause of cancer-related mortality worldwide. In colon cancer, the relationship between age at exposure and excess absolute risk per Gy varies depending on the specific site within the colon [6]. Therefore, a precise evaluation of radiation-related cancer risk requires a detailed understanding of how age at exposure affects cancer risk and the underlying mechanisms in each tissue type.

Animal studies are invaluable for elucidating the mechanisms of radiation-related carcinogenesis and complementing the findings of human epidemiological studies. Studies using *Apc^Min/+^* mice known as human colorectal cancer found that Apc is negatively regulating canonical Wnt signaling as a tumor suppressor gene and demonstrated that infants are more sensitive to radiation-induced intestinal tumors than adults [7,8,9]. This suggests that the mouse gastrointestinal tract is a useful model for investigating mechanisms underlying age-dependent susceptibility to radiation carcinogenesis. As a possible explanation for this age-dependent susceptibility, Miyoshi-Imamura et al. found that infants are more resistant to radiation-induced apoptosis than adults, implying that radiation-induced damaged cells may be eliminated more efficiently in adults than in infants [10]. Moreover, our previous studies showed that the tumorigenicity of *Apc*-deficient stem cells varies with age and is higher in infant mice than in adult mice [9]. As surviving stem cells may serve as the origin of cancer, age-dependent differences in radiation-induced DNA damage responses in intestinal stem and progenitor cells are crucial to investigate.

Recent studies have confirmed the presence of a stem cell population expressing the Lgr5 protein interspersed with Paneth cells in an alternating pattern at the base of mouse intestinal crypts, which are actively dividing. The stem cell marker Lgr5 is a leucin-rich repeat-containing G protein-coupled receptor that enhances Wnt signaling [11,12,13]. A stable pool of Lgr5+ intestinal stem cells is essential for maintaining intestinal homeostasis and radiation-induced intestinal regeneration [14,15,16,17,18,19]. Additionally, p53, a well-known master regulator of stress responses [20], undergoes dynamic changes in the intestinal crypt following radiation exposure [18,21]. Much of this evidence has been derived from studies involving exposure to high doses of radiation and/or conducted in adulthood [22,23], with limited information available on radiation-induced DNA damage responses in the intestinal stem cells of infant mice.

In the present study, we examined the age-dependent radiation responses of stem, progenitor, and differentiated cells in mouse intestinal crypts to uncover the molecular mechanisms underlying age-related radiation effects. We found that the apoptotic cell number and the mitotic index were higher after radiation exposure in the progenitor and differentiated cells of adult intestinal crypts. RNA-sequencing (RNA-seq) analysis revealed that radiation-induced cellular responses, such as decreased expression of genes involved in the cell cycle and DNA repair mechanisms, were observed in adult stem cells but not in infant stem cells.

## 2. Results

### 2.1. Radiation-Induced Apoptosis in Intestinal Crypts as a Function of Age

**Figure 1:**
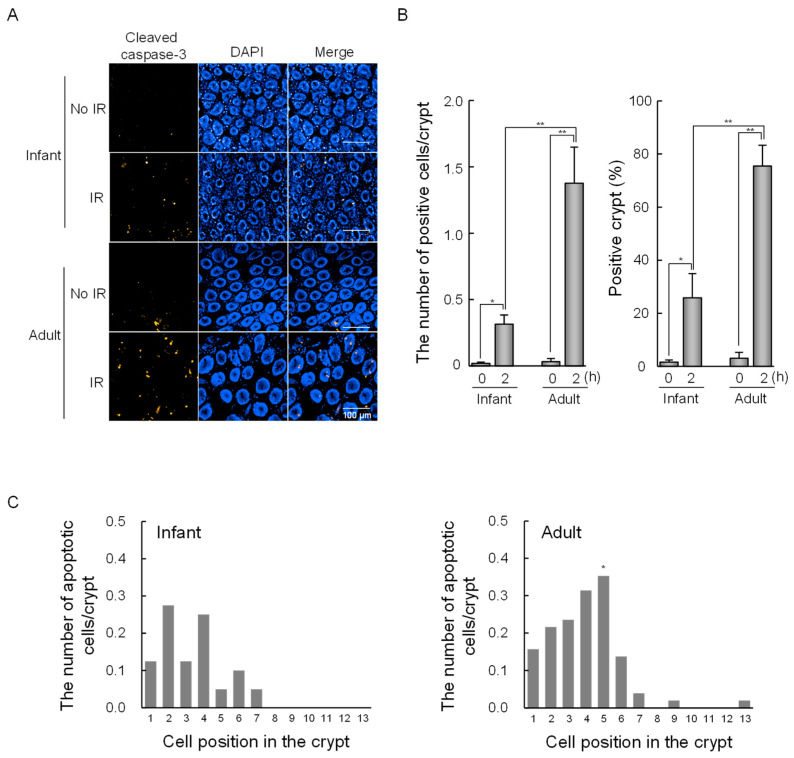
Age-dependent apoptosis after radiation exposure in intestinal crypts from wild-type mice. (**A**) Representative immunofluorescence images stained with cleaved caspase-3 of intestinal crypts 2 h after irradiation. Scale bars: 100 μm. (**B**) Number of apoptotic cells per crypt (**left**) and percentage of apoptotic crypts (**right**). Data are presented as means ± *SD* from three mice. Experiments were performed in duplicates. A one-way ANOVA was used to evaluate differences in means among groups. * *p* < 0.05, ** *p* < 0.01. (**C**) The number of apoptotic cells per crypt was plotted as a function of cell position from the crypt base. Fisher’s exact test was used to evaluate differences in the proportion of cells undergoing apoptosis between infants and adults at different positions from each crypt. * *p* < 0.05.

In our previous study, we explored the age-dependent differences in radiation-induced intestinal tumorigenesis in *Apc^Min/+^* mice exposed to gamma-irradiation at 2 Gy. Thus, to explore age-dependent differences in radiation-induced DNA damage responses in intestinal stem cells, we investigated radiation-induced apoptosis in the intestinal crypts of infant (2 weeks old) and adult (8 weeks old) mice exposed to 2 Gy. Apoptotic cells were defined as those containing apoptotic bodies and exhibiting cleaved caspase-3 staining as determined by three-dimensional (3D) image analysis (Figure 1A). As shown in Figure 1B, the number of apoptotic cells per crypt and the percentage of apoptotic crypts increased at 2 h after irradiation in infants and adults, with significantly higher values observed in adults than in infants (Figure 1B). Interestingly, we observed a clear difference in the locations where apoptosis occurred along the length of the small intestinal crypts (Figure 1C). In adult mouse small intestinal crypts, irradiation induced a high frequency of apoptosis around cell position 5. By contrast, in infant crypts, the frequency of irradiation-induced apoptosis was lower, and apoptotic cells were broadly distributed without a specific location at the base of the crypts.

**Figure 2:**
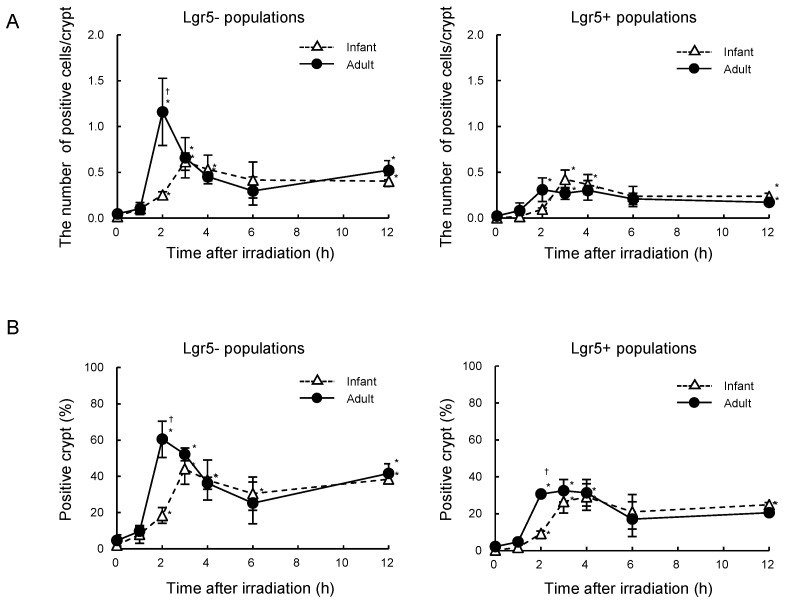
Age-dependent apoptosis after radiation exposure in intestinal crypts from *Lgr5-eGFP-ires*-*Cre^ERT2^* mice. (**A**) Number of cleaved caspase-3 positive cells per crypt as a function of time after irradiation. Data are from Lgr5- populations (**left**) and Lgr5+ populations (**right**) in the intestinal crypt. Data are presented as means ± *SD* from three mice. Experiments were performed in duplicates. A one-way ANOVA was used to evaluate differences in means among groups. * *p* < 0.05 vs. the non-irradiated group. ^†^ *p* < 0.05 vs. the group at the same time point after irradiation. (B) Percentage of crypts containing cleaved caspase-3 positive cells as a function of time after irradiation. Data are from Lgr5- populations (left) and Lgr5+ populations (right) in the intestinal crypt. Data are presented as means ± *SD* from three mice. Experiments were performed in duplicates. A one-way ANOVA was used to evaluate differences in means among groups. * *p* < 0.05 vs. the non-irradiated group. ^†^ *p* < 0.05 vs. the infant group at the corresponding time point after irradiation.

For further analysis, we used *Lgr5-eGFP-ires-Cre^ERT2^* mice, in which Lgr5-expressing stem cells expressed eGFP, allowing us to analyze Lgr5-expressing stem cells and other progenitor/differentiated cells separately (Supplementary Figure S1). Radiation exposure increased the number of apoptotic cells in the infant and adult crypts, with different patterns (Supplementary Figure S2). In the Lgr5- populations from adult mouse intestinal crypts, the mean number of apoptotic cells peaked sharply at 2 h and then decreased after radiation exposure. Conversely, the number of apoptotic cells in the Lgr5- populations from infants increased more gradually, reaching its peak at 3 h following irradiation (Figure 2A). The apoptotic cell number at 2 h after radiation exposure was significantly higher in adults than in infants. In the Lgr5+ populations, the mean number of apoptotic cells increased modestly but significantly compared with the pre-irradiation after exposure, with a peak occurring at 3–4 h in infants and 2–4 h in adults, respectively. There was no significant difference in the number of radiation-induced apoptotic cells between infants and adults at the corresponding time point in the Lgr5+ populations. A similar trend, in which the peak of apoptosis was higher in adult crypts, was observed in the percentage of crypts containing apoptotic cells in the Lgr5- populations (Figure 2B).

### 2.2. Mitotic Index in Intestinal Crypts after Radiation Exposure as a Function of Age

**Figure 3:**
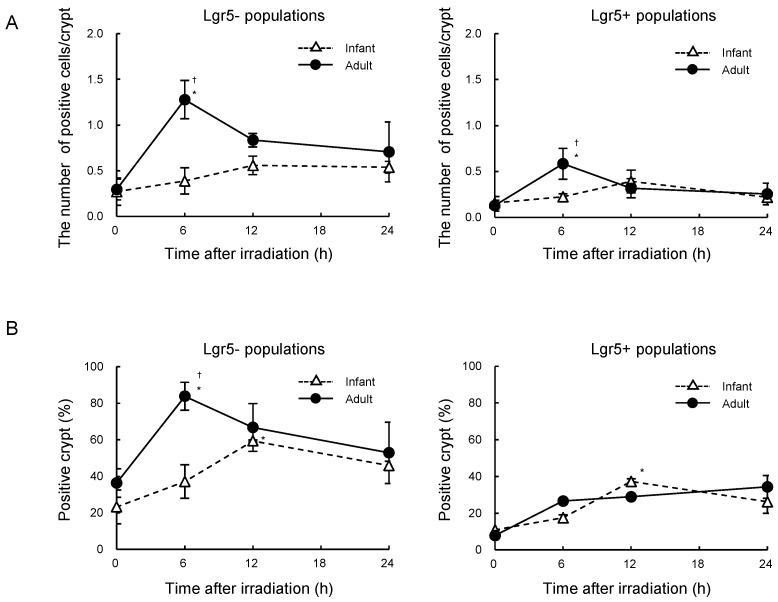
Age-dependent mitotic index after radiation exposure in intestinal crypts from *Lgr5-eGFP-ires*-*Cre^ERT2^* mice. (**A**) Number of phospho-histone H3-positive cells per crypt as a function of time after irradiation. Data are from Lgr5- populations (**left**) and Lgr5+ populations (**right**) in the intestinal crypt. Data are presented as means ± *SD* from three mice. Experiments were performed in duplicates. A one-way ANOVA was used to evaluate differences in means among groups. * *p* < 0.05 vs. the non-irradiated group. ^†^ *p* < 0.05 vs. the infant group at the same time point after irradiation. (**B**) Percentage of crypts containing phospho-histone H3-positive cells as a function of time after irradiation. Data are from Lgr5- populations (**left**) and Lgr5+ populations (**right**) in the intestinal crypt. Data are presented as means ± *SD* from three mice. Experiments were performed in duplicates. A one-way ANOVA was used to evaluate differences in means among groups. * *p* < 0.05 vs. the non-irradiated group. ^†^ *p* < 0.05 vs. the infant group at the corresponding time point after irradiation.

We examined age-dependent mitotic index in the intestinal crypts using phosphorylated histone H3 (Ser10) as a mitotic marker. The number of phospho-histone H3 positive cells per crypt decreased 0.5 h, reached a minimum at 1 h, and subsequently recovered to pre-irradiation level at 4 h post-irradiation both in infant and adult crypts. At 6 h following irradiation, however, the number of phospho-histone H3 positive cells increased significantly in adult crypts but not in infant crypts, irrespective of Lgr5 expression (Figure 3A). The mitotic index at 6 h post-irradiation was notably higher in adults compared to infants, both in Lgr5- and Lgr5+ populations. A similar trend, in which the mitotic index was higher in adult crypts, was observed in the percentage of crypts containing phospho-histone H3-positive cells in the Lgr5- populations (Figure 3B).

### 2.3. p53 Activation after Radiation Exposure as a Function of Age

**Figure 4:**
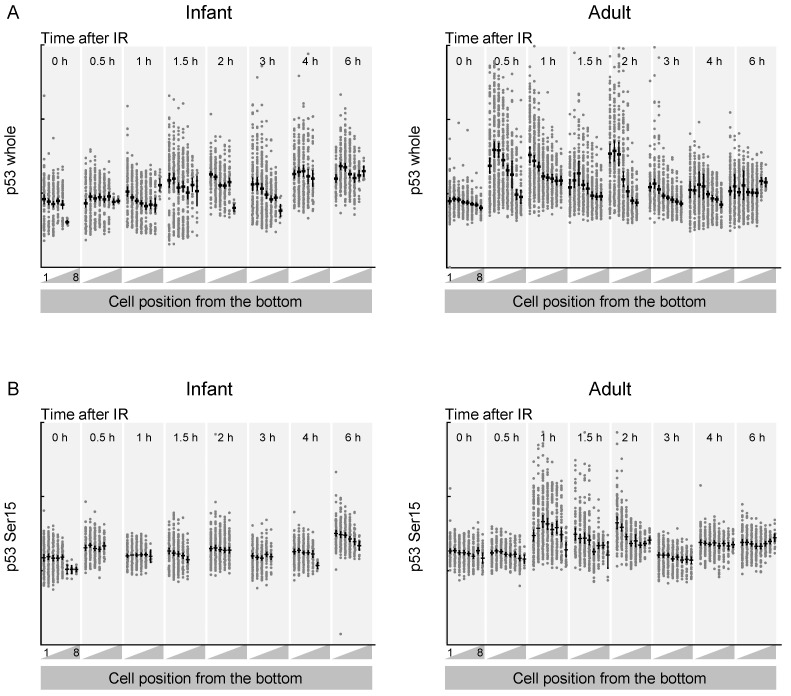
Average p53 immunofluorescence intensity in mouse intestinal crypts as a function of time after irradiation. (**A**) Quantification of p53 intensity across the intestinal crypts of mice treated with irradiation and analyzed at the indicated time points. (**B**) Quantification of phospho-p53 intensity across the intestinal crypts of mice treated with irradiation and analyzed at the indicated time points. (**A**,**B**) Data are from infant (**left**) and adult (**right**) mice. Bold bar shows mean and 95% confidential intervals. Dots represent individual cells. Experiments were performed in duplicates.

Previous studies have reported that the temporal dynamics of p53 play a crucial role in determining cell fate following DNA damage [20,21,24]. To quantify p53 activation, we stained whole-mount mouse intestinal tissue with antibodies against p53. Minimal p53 staining was observed in the tissues of untreated mice (Figure 4A). Following radiation exposure, p53 staining was not uniformly distributed throughout the intestine but showed strong localization in crypt cells. In adult crypts, p53 levels increased at 0.5 h after irradiation and returned to near-background levels by 6 h. By contrast, in infant crypts, p53 levels exhibited a slower and less pronounced increase after exposure to radiation. Phospho-p53, an activated form produced by DNA damage-responsive kinases such as ATM, showed similar age-specific dynamics [20]. Phospho-p53 levels increased one hour after irradiation in adult crypts but not in infant crypts (Figure 4B). Collectively, these results demonstrate that the dynamics of the p53 response vary with age.

### 2.4. Gene Expression Profile in Lgr5+ Stem Cells after Radiation Exposure as a Function of Age

**Figure 5:**
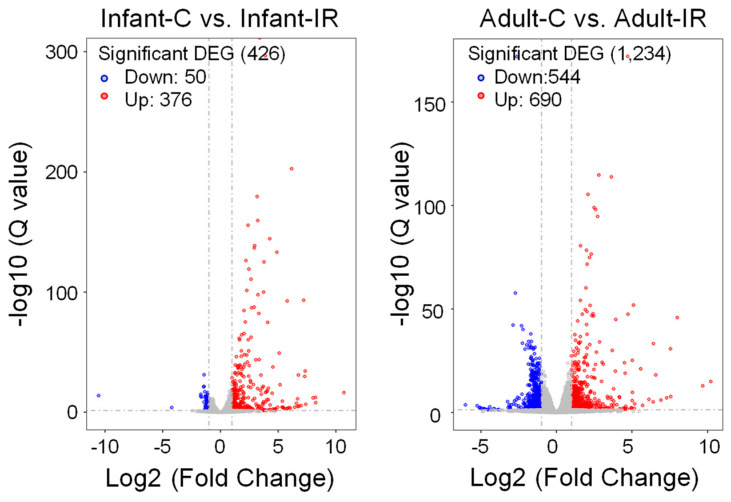
Volcano plot of DEGs between non-irradiated and irradiated groups from infant (**left**) and adult (**right**) stem cell populations. Red dots, upregulated DEGs; blue dots, downregulated DEGs; grey dots, nonsignificant DEGs.

**Figure 6:**
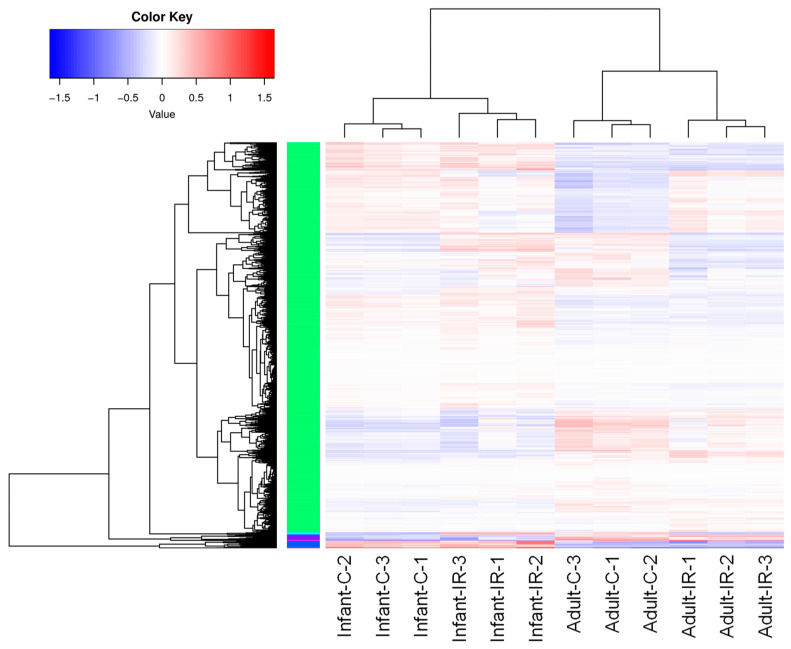
Heatmap of DEGs from the infant non-irradiated (Infant-C), infant irradiated (Infant-IR), adult non-irradiated (Adult-C), and adult irradiated (Adult-IR) groups. The color keys of blue (**low**), white (**medium**), and red (**high**) represent the express levels of different genes. Their expression patterns can be classified mainly into six clusters.

To investigate the radiation response in intestinal stem cells, we isolated Lgr5+ stem cell populations and performed RNA sequencing (RNA-seq) to compare age-related changes in gene expression induced by radiation exposure. Supplementary Figure S3 shows a volcano plot of differentially expressed genes (DEGs) between infants and adults without or with radiation exposure (left: infant non-irradiated (Infant-C) group vs. adult non-irradiated (Adult-C) group, right: infant irradiated (Infant-IR) group vs. adult irradiated (Adult-IR) group). As shown in Figure 5, RNA-seq analysis results revealed 426 DEGs (greater than 2-fold change, *p* < 0.05), of which 376 were upregulated and 50 were downregulated in the irradiated infant group compared with the non-irradiated infant group. In adult mouse intestinal stem cells, we observed extensive changes in DEGs, with 690 upregulated and 544 downregulated genes, following irradiation. Hierarchical clustering was performed to group genes and samples based on their expression profiles (Figure 6). This analysis revealed a distinct separation between the infant and adult samples regardless of the irradiation status. Within each age group, the samples were further clustered into nonirradiated and irradiated groups. Principal component analysis (PCA) of the DEG expression patterns highlighted this separation (Supplementary Figure S4).

**Figure 7:**
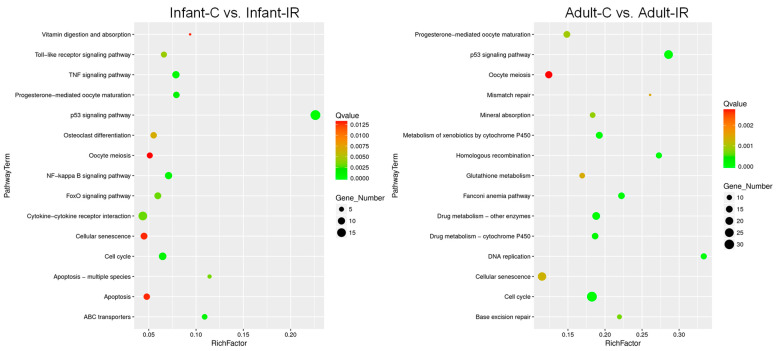
Bubble charts of the KEGG classifications of assembled DEGs. Top 15 enriched KEGG pathway analysis on DEGs after irradiation in infant (**left**) and adult (**right**) stem cell populations. The number of DEGs enriched in the pathway is indicated by circle size. The Rich factor is the ratio of the number of DEGs annotated in a pathway to the number of all genes annotated in this pathway. The color saturation from green to red indicates the Q value.

To further characterize the functional roles of DEGs in infant and adult intestinal stem cell populations after irradiation, we conducted a GO enrichment analysis and a pathway-based analysis using the Kyoto Encyclopedia of Genes and Genomes (KEGG) pathway database (https://www.genome.jp/kegg/, accessed on 14 August 2024). Regardless of radiation exposure, the DEGs in the GO-enriched category of biological processes were mainly involved in responses to bacteria, such as defense responses to gram-positive and -negative bacteria (Supplementary Figure S5). The KEGG enrichment analysis also showed that similar pathways were enriched between infants and adults, regardless of radiation exposure (Supplementary Figure S6). As illustrated in Figure 7, KEGG enrichment analysis results indicated that the p53 signaling pathway, cell cycle, cellular senescence, and oocyte meiosis were enriched following radiation exposure in the infant and adult samples (Figure 7 and Table 1). Notably, in the cell cycle pathway, more downregulated DEGs were found across the G1, S, G2, and M phases in the adult Lgr5+ stem cells following irradiation. Interestingly, DNA repair pathways, such as homologous recombination, Fanconi anemia, base excision repair, and mismatch repair, were also significantly enriched in the DEGs. These cell cycle pathways and DNA repair pathways were not enriched in the DEGs from Infant-C vs. Adult-C and Infant-IR vs. Adult-IR. These suggest that DNA damage responses such as cell cycle arrest and DNA repair were activated in adult intestinal Lgr5+ stem cell populations after radiation exposure, not solely dependent on age.

## 3. Discussion

In this study, we investigated the age-dependent radiation-induced DNA damage responses in stem, progenitor, and differentiated cells from mouse intestinal crypts. We found that the apoptotic cell number and the mitotic index were significantly higher after radiation exposure in the adult Lgr5- cells than in the adult Lgr5+ stem cells and infant Lgr5-/Lgr5+ cells. Supporting these findings, more dynamic radiation-induced p53 activity was observed in the adult Lgr5+ stem cells. RNA-seq analysis revealed more DEGs involved in cell cycle regulation and DNA repair following radiation exposure in adult stem cells than in infant stem cells, not depending on age. These results suggest that adult mouse intestinal crypts have developed more robust protective mechanisms against radiation exposure than infant mouse intestinal crypts, potentially resulting in a lower susceptibility to radiation carcinogenesis.

Animal studies have provided insights into the age-dependence of radiation carcinogenesis, demonstrating a susceptible age window for radiation-induced tumorigenesis depending on the organ [49,50,51,52,53,54]. These evidences imply that the difference in the viability of stem and progenitor cells after irradiation is a common explanation for the age-dependent susceptibility to radiation-induced carcinogenesis. In mouse small intestinal crypts, radiation-induced apoptosis is less frequent in infants, who are more susceptible to radiation carcinogenesis than adults [7,8,9]. By contrast, adult mice are more susceptible to radiation-induced myeloid leukemia, which arises from hematopoietic stem and progenitor cells, than infant mice [55,56]. Ariyoshi et al. demonstrated that the hematopoietic stem and progenitor cells in infant mice are more radiosensitive than those in adult mice [50]. Exposure during puberty poses the greatest risk in rat mammary cancers [52,54]. The radiosensitivity of rat mammary clonogens, defined as cells capable of clonal growth, is lower at the onset of puberty (between 4 and 6 weeks of age) than the prepubertal period [57]. These data suggest that radiation-induced cell death, including apoptosis, may be a modifying factor that contributes to age-dependent susceptibility to radiation tumorigenesis by eliminating oncogenic stem cells.

In mice, intestinal architecture is not fully developed at birth; newborn mice possess villi but lack crypts [9,58,59]. After birth, crypts begin to emerge and undergo continuous elongation and multiplication through crypt fission for a short period until weaning [60,61,62,63]. We previously reported that the postnatal period before weaning, when the frequency of crypt fission is most prevalent, is most susceptible to radiation carcinogenesis [9]. Itzkovitz et al. proposed a theoretical and experimental model in which the initial expansion of the entire stem cell pool occurs via symmetric stem cell divisions, followed by a sharp transition to non-stem cell production through asymmetric divisions, minimizing the time required to form a mature crypt during the prenatal stage [64]. We postulate that the stem cell renewal, including symmetric and asymmetric division, and the surrounding environment during crypt fission affect the radiation-induced DNA damage responses in infant mice. Our RNA-seq data revealed that different p53 signaling pathways were activated by irradiation in infants and adults. p53 orchestrates symmetric and asymmetric self-renewal of stem cells not only at a functional level but also at a genomic level [65,66]. Stem cells and their surrounding microenvironment communicate through mechanical cues to regulate stem cell behavior during crypt fission [67,68,69]. In this process, local clusters of relatively soft intestinal crypt stem cells are particularly susceptible to deformation in response to mechanical forces. These invaginating stem cell clusters subsequently expand into the lumen, ultimately leading to the division of the original stem cell niche. Recently, the study of physical and mechanical features of stem cells has become a new frontier in cancer research and stem cell biology [68,69,70,71,72,73,74]. Further studies will elucidate the biochemical and biophysical factors involved in the interaction between stem cells and the surrounding cells during crypt fission, as well as the regulatory pathways influencing susceptibility to radiation-induced tumorigenesis.

In the present study, while radiation increased *p53* mRNA and protein expression in adult intestinal stem cells, it only increased *p53* mRNA expression in infant intestinal stem cells. This discrepancy between the gene and protein expression of p53 in infants may be due to the negative feedback regulation of p53. Mdm2 controls p53 transcriptional activity by regulating p53 protein stability, whereas MdmX (Mdm4) functions as a p53 transcriptional inhibitor without altering p53 levels [20,75]. Wip1 (*Ppm1d*) inactivates p53 in several ways [20,75]. Additionally, Wip1 (*Ppm1d*) can inactivate p53 function through several mechanisms [19,42]. As shown in Supplementary Figure S7, the expression levels of Mdm2 and Mdm4 were upregulated after radiation exposure in the infant and adult stem cells. Interestingly, Ppm1d was upregulated by irradiation in infant stem cells but not in adult stem cells. These DEGs may affect the p53 protein dynamics in infant and adult stem cells following radiation exposure. Further analyses, such as comprehensive multi-omics single-cell data integration, including genomic single-cell data, are expected to elucidate the mechanisms of radiation-induced DNA damage response and its regulatory integration.

In summary, our data suggest that radiation-induced DNA damage responses in mouse intestinal crypts differ between infants and adults. This result may be related to the age-dependent susceptibility to radiation carcinogenesis. We hypothesized that the mechanisms of radiation-induced tumorigenesis differ depending on age at exposure. Understanding how age influences the susceptibility of specific organs to radiation-induced carcinogenesis and uncovering the underlying mechanisms are essential for radiation protection.

## 4. Materials and Methods

### 4.1. Mice

C57BL/6 (B6) and B6-*Lgr5-ires-eGFP-Cre^ERT2^* mice were purchased from The Jackson Laboratory (Catalog #: 008875, Bar Harbor, ME, USA). At least three mice were used at each time point, and the experiment was conducted twice. This study was conducted in accordance with the Guide for the Care and Use of Laboratory Animals of the Hiroshima University Animal Research Committee. The protocol was approved by the Committee on Ethics of Animal Experiments at Hiroshima University. All mice were maintained in accordance with the guidelines of the Institute of Laboratory Animal Science of Hiroshima University, and efforts were exerted to minimize suffering.

### 4.2. Irradiation

Mice were irradiated with an acute dose of 2 Gy gamma rays using a Gamma Cell 40 Exactor Research Irradiator equipped with a 148 TBq ^137^Cs source (Best Theratronics, Ottawa, ON, Canada). The irradiation was performed at a dose rate of 770 mGy/min. The total absorbed dose was calibrated using a GD-302M glass dosimeter (AGC Techno Glass Co., Ltd., Shizuoka, Japan).

### 4.3. Immunofluorescent Staining of 3D Whole-Mount Tissue

The mice were euthanized by CO_2_ exposure, and the intestines were immediately removed. Intestinal tissues (0.5 cm × 0.5 cm) were fixed with 4% paraformaldehyde in PBS (pH 7.4) overnight at 4 °C. The samples were immersed in the tissue-clearing reagent CUBIC-L (Tokyo Chemical Industry, Tokyo, Japan) overnight, permeabilized with 1% Triton X-100 in PBS for 2 h, and then incubated in a blocking solution for 2 h (1% bovine serum albumin, 3% normal goat serum, and 0.2% Triton X-100 in PBS). Primary antibodies were diluted in Can Get Signal™ Immunostain Immunoreaction Enhancer Solution B (TOYOBO, Osaka, Japan) as follows: anti-Cleaved Caspase-3 (Asp175) (Cell Signaling Technology, Danvers, MA, USA) and anti-phospho-Histone H3 (Ser10) (Mitotic Marker) (Cell Signaling Technology, Danvers, MA, USA). The samples were incubated with primary antibodies for 1 day at 4 °C. They were then washed three times with PBS-T (0.1% Tween^®^20 in PBS) and incubated with the secondary antibody anti-rabbit Alexa Fluor 555 (Thermo Fisher, Tokyo, Japan) and counterstained with DAPI (1 μM) (Merck, Tokyo, Japan) overnight at 4 °C. The specimens were transferred to 96-well plates and immersed in tissue-clearing reagent CUBIC-R (Tokyo Chemical Industry, Tokyo, Japan) for image acquisition. Automated imaging was performed using a high-content screening system, Opera Phenix^®^ (PerkinElmer Japan G.K., Kanagawa, Japan), with a 20× 1.0 NA water objective in confocal optical mode, and Z-stacks were taken at 0.8 μm intervals. Images were analyzed using the Harmony software package, version 4.9. (PerkinElmer Japan G.K., Kanagawa, Japan).

### 4.4. Isolation of Single Crypt Cells from Mouse Intestinal Tissue

Intestinal crypts were isolated following the protocol described by Zaharieva et al., with modifications. A 10 cm segment of the small intestine was cut into 2–3 mm pieces and washed with ice-cold HBSS(-)/antibiotics [76]. Tissue fragments were incubated in 30 mM EDTA/HBSS(-) for 5 min at 25 °C to dissociate the crypts from the intestinal tissue. The tissue was passed through a 70 μm cell strainer, washed with ice-cold HBSS(-)/antibiotics/2% FBS, and subjected to enzymatic digestion in collagenase D (Roche, Mannheim, Germany)/dispase (STEMCELL Technologies, Toronto, ON, Canada) for 30 min at 37 °C in a shaking water bath at 200 rpm. The digested intestinal tissue was passed through a 40 μm cell strainer and suspended in PBS(-)/antibiotics/2% FBS. The cells were stained with 7-AAD (BD Biosciences, Tokyo, Japan) and isolated using a FACS Aria (BD Biosciences, Tokyo, Japan) and FACS SORP (BD Biosciences, Tokyo, Japan).

### 4.5. RNA-Seq and Analysis

Total RNA was extracted using the RNeasy Mini Kit (QIAGEN, Hilden, Germany). RNA sequencing (RNA-seq) was performed by GENEWIZ, Inc. (Tokyo, Japan) using a next-generation sequencer (NovaSeq, Illumina, San Diego, CA, USA). The generated sequence tags (more than 4.0 × 10^7^ reads for each sample) were mapped to the mouse genomic sequence (Genome assembly GRCm39) using the sequence alignment program HISAT2 (v2.2.1). Transcriptomes were compared using FPKM values and analyzed with the KEGG database (https://www.genome.jp/kegg/, accessed on 14 August 2024) to identify the number of DEGs in each pathway. Additionally, KEGG enrichment analyses were performed to identify significantly enriched KEGG terms among the DEGs.

### 4.6. Statistical Analysis

The results are presented as mean ± SD. Differences between groups were evaluated through one-way ANOVA using the GraphPad Prism software package, version 8.2.0 and *t*-tests using the StatMate III software package (https://atms.jp/, accessed on 2 August 2024). We tested the proportion of cells undergoing apoptosis between infants and adults at different positions from each crypt using Fisher’s exact test. A *p* value of <0.05 was considered statistically significant.

## Figures and Tables

**Table 1:** Pathway analysis showing top associations with genes whose expression levels were changed by irradiation.

Pathway ID	Pathway	Upregulated Genes	Downregulated Genes	*p* Value	Q Value
Infant-C vs. Infant-IR					
ko04115	p53 signaling pathway [24]	*Ccng1*, *Sesn2*, *Mdm2*, *Zmat3*, *Tnfrsf10b*, *Bbc3*, *Gtse1*, *Pidd1*, *Cdkn1a*, *Bax*, *Apaf1*, *Mdm4*, *Ei24*, *Ppm1d*, *Fas*, *Igfbp3*, *Rprm*	*Ccnb1*, *Ccnb2*	4.9 × 10^−18^	1.3 × 10^−15^
ko04668	TNF signaling pathway [25]	*Lif*, *Icam1*, *Jag1*, *Csf1*, *Jag2*, *Fas*, *Tnf*, *Map3k8*, *Ccl20*, *Nod2*,		9.2 × 10^−6^	4.6 × 10^−4^
ko04110	Cell cycle [26]	*Mdm2*, *Cdkn1a*, *Tgfb1*	*Plk1*, *Cdc20*, *Ccnb1*, *Cdkn2c*, *Dbf4*, *Cdc25c*, *Ccnb2*, *Ccna2*	2.9 × 10^−5^	8.0 × 10^−4^
ko04064	NF-kappa B signaling pathway [27]	*Pidd1*, *Icam1*, *Nfkb2*, *Eda2r*, *Relb*, *Tnf*, *Cd40*, *Cd14*, *Plau*, *Tlr4*		2.5 × 10^−5^	8.0 × 10^−4^
ko02010	ABC transporters [28]	*Tap1*, *Abcb1b*, *Abcc5*, *Abcc4*, *Abca1*, *Abca8b*		3.5 × 10^−5^	8.3 × 10^−4^
ko04914	Progesterone-mediated oocyte maturation [29]	*Adcy2*	*Kif22*, *Plk1*, *Aurka*, *Ccnb1*, *Cdc25c*, *Ccnb2*, *Ccna2*	4.9 × 10^−5^	1.1 × 10^−3^
ko04060	Cytokine-cytokine receptor interaction [30]	*Gdf15*, *Tnfrsf10b*, *Lif*, *Eda2r*, *Csf1*, *Fas*, *Tnf*, *Tnfsf4*, *Tnfrsf18*, *Cd40*, *Tnfrsf21*, *Tnfrsf19*, *Ccl20*, *Tgfb1*, *Tnfrsf11b*		2.2 × 10^−4^	3.0 × 10^−3^
ko04068	FoxO signaling pathway [31]	*Mdm2*, *Cdkn1a*, *Plk1*, *Homer3*, *Prkag3*, *Tgfb1*	*Plk2*, *Ccnb1*, *Ccnb2*	2.3 × 10^−4^	3.0 × 10^−3^
ko04215	Apoptosis—multiple species [32]	*Bbc3*, *Bax*, *Apaf1*	*Birc5*	2.7 × 10^−4^	3.3 × 10^−3^
ko04620	Toll-like receptor signaling pathway [33]	*Cd80*, *Tnf*, *Map3k8*, *Cd40*, *Cd14*, *Ikbke*, *Tlr4*		4.1 × 10^−4^	4.1 × 10^−3^
ko04380	Osteoclast differentiation [34]	*Nfkb2*, *Csf1*, *Relb*, *Nfatc2*, *Tnf*, *Sirpa*, *Tgfb1*, *Tnfrsf11b*		7.5 × 10^−4^	6.6 × 10^−3^
ko04210	Apoptosis [32]	*Tnfrsf10b*, *Bbc3*, *Pidd1*, *Bax*, *Apaf1*, *Fas*, *Tnf*	*Birc5*	2.0 × 10^−3^	1.3 × 10^−2^
ko04218	Cellular senescence [26,35]	*Mdm2*, *Cdkn1a*, *Nfatc2*, *Ccnb1*, *H2-M2*, *Igfbp3*, *Ccnb2*, *Ccna2*, *Tgfb1*		2.0 × 10^−3^	1.3 × 10^−2^
ko04977	Vitamin digestion and absorption [36]	*Slc19a2*, *Wdr91*, *Cubn*		1.9 × 10^−3^	1.3 × 10^−2^
ko04114	Oocyte meiosis [37,38]	*Adcy2*	*Plk1*, *Cdc20*, *Aurka*, *Ccnb1*, *Cdc25c*, *Ccnb2*	2.2 × 10^−3^	1.3 × 10^−2^
Adult-C vs. Adult-IR					
ko04115	p53 signaling pathway [24]	*Ccng1*, *Cdkn1a*, *Zmat3*, *Mdm2*, *Sesn2*, *Tnfrsf10b*, *Bax*, *Mdm4*, *Gtse1*, *Bbc3*, *Fas*, *Pidd1*, *Igfbp3*, *Gadd45a*, *Adgrb1*	*Ccnb2*, *Ccnb1*, *Cdk1*, *Sesn3*, *Ccne2*, *Cdk6*, *Chek1*, *Ccne1*, *Serpine1*	2.6 × 10^−13^	8.6 × 10^−11^
ko04110	Cell cycle [26]	*Cdkn1a*, *Mdm2*, *Gadd45a*, *Cdkn2b*	*Bub1b*, *Ccna2*, *Ccnb2*, *Plk1*, *Dbf4*, *Cdc20*, *Bub1*, *Espl1*, *Ccnb1*, *Mad2l1*, *Cdc25c*, *Ttk*, *Orc1*, *Cdk1*, *Cdc6*, *Cdc45*, *Ccne2*, *Cdk6*, *Chek1*, *Ccne1*, *Cdkn2c*, *Skp2*, *Cdc7*, *Mcm5*, *Mcm6*, *Mcm4*, *Mcm3*	9.7 × 10^−11^	1.6 × 10^−8^
ko03030	DNA replication [39]		*Rfc3*, *Fen1*, *Rpa1*, *Pold3*, *Rpa2*, *Prim2*, *Pole2*, *Mcm5*, *Mcm6*, *Mcm4*, *Lig1*, *Mcm3*	1.1 × 10^−8^	1.2 × 10^−6^
ko03440	Homologous recombination [40]		*Rad54b*, *Eme1*, *Rpa1*, *Pold3*, *Brca1*, *Rpa2*, *Rad51b*, *Rad54l*, *Bard1*, *Rad51c*, *Xrcc2*, *Blm*	1.6 × 10^−7^	1.1 × 10^−5^
ko00983	Drug metabolism—other enzymes [41]	*Ces2b*, *Mgst2*, *Ugt2b5*, *Ces2f*, *Ugt2b36*, *Tymp*, *Gsta13*, *Ces1f*, *Gsta2*, *Gsta3*, *Gsta5*, *Ces2e*, *Gsta1*, *Ces1c*, *Gsta4*, *Ces2h*	*Cyp2e1*, *Tk1*, *Rrm1*	1.4 × 10^−7^	1.1 × 10^−5^
ko03460	Fanconi anemia pathway [42]	*Polk*, *Rev1*, *Dennd2a*	*Fancb*, *Usp1*, *Eme1*, *Rpa1*, *Brca1*, *Rpa2*, *Fancd2*, *Fancg*, *Cenps*, *Rad51c*, *Blm*	4.2 × 10^−7^	2.3 × 10^−5^
ko00980	Metabolism of xenobiotics by cytochrome P450 [43]	*Ephx1*, *Mgst2*, *Ugt2b5*, *Gstk1*, *Ugt2b36*, *Gsta13*, *Cbr3*, *Gsta2*, *Gsta3*, *Gsta5*, *Gsta1*, *Gsta4*, *Adh4*, *Adh7*	*Cyp2e1*	1.5 × 10^−6^	5.6 × 10^−5^
ko00982	Drug metabolism—cytochrome P450 [44]	*Mgst2*, *Ugt2b5*, *Gstk1*, *Ugt2b36*, *Gsta13*, *Gsta2*, *Gsta3*, *Gsta5*, *Gsta1*, *Gsta4*, *Fmo5*, *Adh4*, *Adh7*	*Cyp2e1*	4.5 × 10^−6^	1.2 × 10^−4^
ko03410	Base excision repair [45]		*Neil3*, *Hmgb1*, *Fen1*, *Pold3*, *Mbd4*, *Ung*, *Mutyh*, *Pole2*, *Lig1*	2.9 × 10^−5^	6.9 × 10^−4^
ko04978	Mineral absorption [46]	*Slc6a19*, *Slc26a3*, *Slc34a2*, *Slc5a1*, *Slc26a6*, *Hmox1*, *Mt2*, *Mt1*, *Slc9a3*	*Atp1b2*, *Cybrd1*	4.0 × 10^−5^	8.3 × 10^−4^
ko04914	Progesterone-mediated oocyte maturation [47]		*Kif22*, *Ccna2*, *Ccnb2*, *Plk1*, *Bub1*, *Aurka*, *Ccnb1*, *Mad2l1*, *Cdc25c*, *Cdk1*, *Pde3b*, *Prkacb*, *Adcy8*, *Rps6ka6*, *Pgr*	4.8 × 10^−5^	9.2 × 10^−4^
ko04218	Cellular senescence [26,35]		*Cdkn1a*, *Mdm2*, *Foxm1*, *Ccna2*, *Ccnb2*, *Ccnb1*, *Cdk1*, *Nfatc2*, *Ccne2*, *Cdk6*, *Chek1*, *Ccne1*, *H2-T25*, *Igfbp3*, *Gadd45a*, *Cacna1d*, *H2-Q1*, *Mras*, *Cdkn2b*, *Serpine1*, *Rassf5*, *H2-Q7*, *H2-T27*	8.1 × 10^−5^	1.3 × 10^−3^
ko00480	Glutathione metabolism [41]	*Mgst2*, *Ggt1*, *Gstk1*, *Gsta13*, *Anpep*, *Gsta2*, *Gsta3*, *Gsta5*, *Gsta1*, *Rrm1*, *Gsta4*		9.2 × 10^−5^	1.4 × 10^−3^
ko03430	Mismatch repair [48]		*Rfc3*, *Rpa1*, *Pold3*, *Rpa2*, *Exo1*, *Lig1*	1.0 × 10^−4^	1.5 × 10^−3^
ko04114	Oocyte meiosis [37]	*Adcy8*	*Ccnb2*, *Plk1*, *Cdc20*, *Bub1*, *Espl1*, *Aurka*, *Ccnb1*, *Mad2l1*, *Cdc25c*, *Cdk1*, *Sgo1*, *Ccne2*, *Ccne1*, *Prkacb*, *Rps6ka6*, *Pgr*	2.0 × 10^−4^	2.7 × 10^−3^

## Data Availability

The data presented in this study are available upon request from the corresponding author.

## Supplementary Materials

The following supporting information can be downloaded at https://www.mdpi.com/article/10.3390/ijms251810213/s1.
