# Supplementary material for: Age-Dependent Differences in Radiation-Induced DNA Damage Responses in Intestinal Stem Cells
Source: Int J Mol Sci. 2024 Sep 23;25(18):10213. doi: 10.3390/ijms251810213 (PMC11431935; doi:10.3390/ijms251810213)
Supplement: Supplementary file 1 [file ijms-25-10213-s001.zip › ijms-3204816-supplementary.pdf]

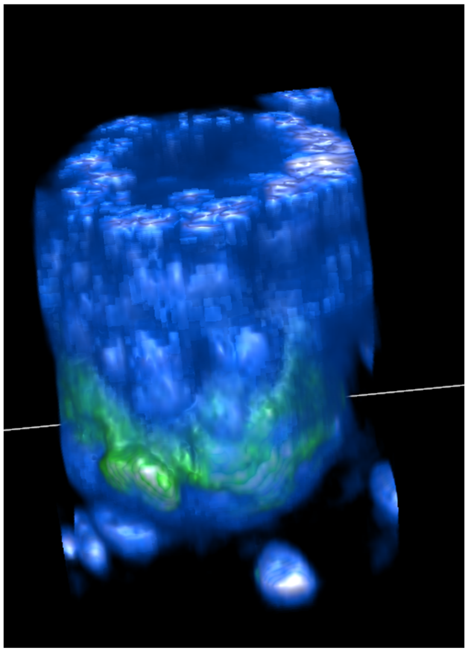

Supplementary Figure S1. Immunofluorescence images of intestinal crypts in 3D by using *Lgr5-eGFP-ires-Cre^{ERT2}* mouse. Intestine was stained with DAPI (blue); Lgr5-positive (GFP-positive) stem cells shown in green.

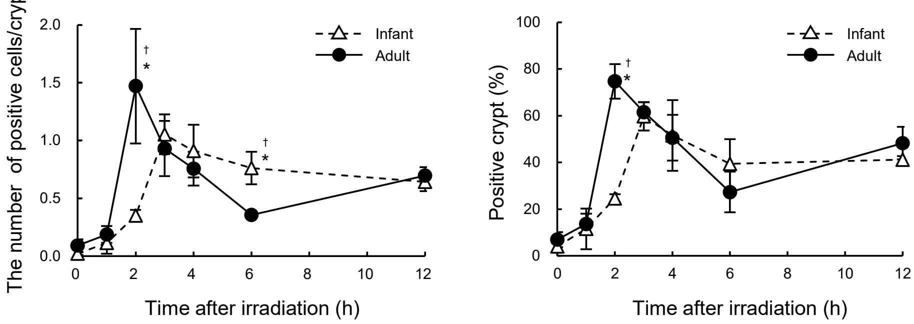

Supplementary Figure S2. Age-dependent apoptosis after radiation exposure in intestinal crypts from *Lgr5-eGFP-ires-Cre*^{ERT2} mice. Number of cleaved caspase-3 positive cells per crypt as a function of time after irradiation (left). Percentage of crypts containing cleaved caspase-3 positive cells as a function of time after irradiation. Data are presented as means ± *SD* from three mice. Experiments were performed in duplicates. One-way ANOVA was used to evaluate differences in means among groups. *$p<0.05$ vs. the non-irradiated group. †$p<0.05$ vs. the infant group at the corresponding time point after irradiation.

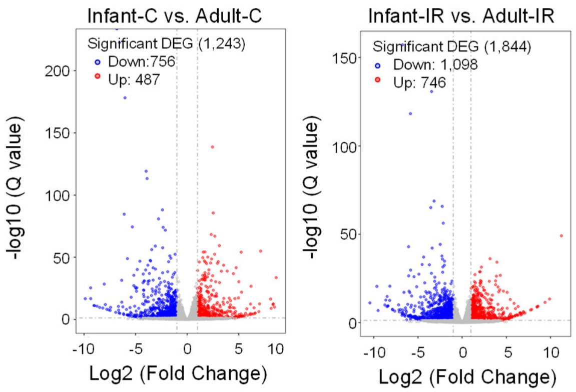

Supplementary Figure S3. Volcano plot of DEGs between the non-irradiated infant and non-irradiated adult groups (left) and between the irradiated infant and irradiated adult groups (right). Red dots, upregulated DEGs; blue dots, downregulated DEGs; and gray dots, non-significant DEGs.

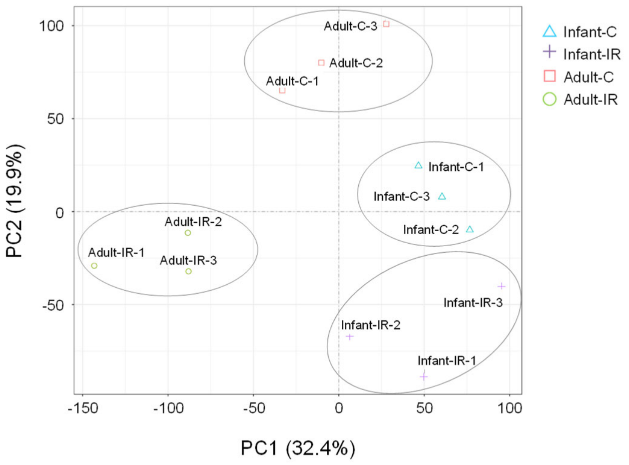

Supplementary Figure S4. PCA chart of RNA-seq data. Each point represents an RNA-Seq sample. Samples with similar gene expression profiles are clustered together. Sample groups are indicated by using different colors as indicated in the legend provided.

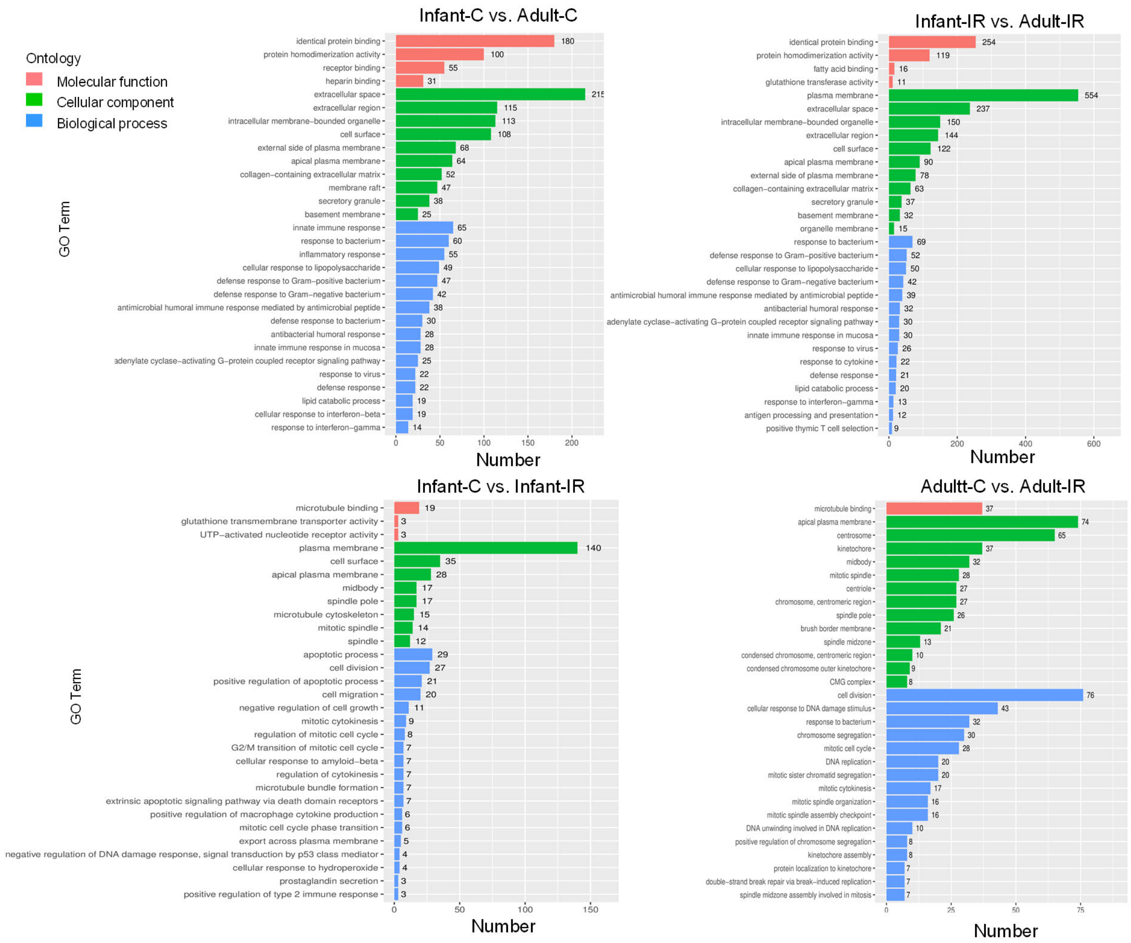

Supplementary Figure S5. Gene Ontology (GO) term enrichment analysis. Significantly enriched GO terms were selected based on an FDR < 0.05. GO terms of the categories of molecular functions, cellular components, and biological processes are depicted in red, green, and blue, respectively.

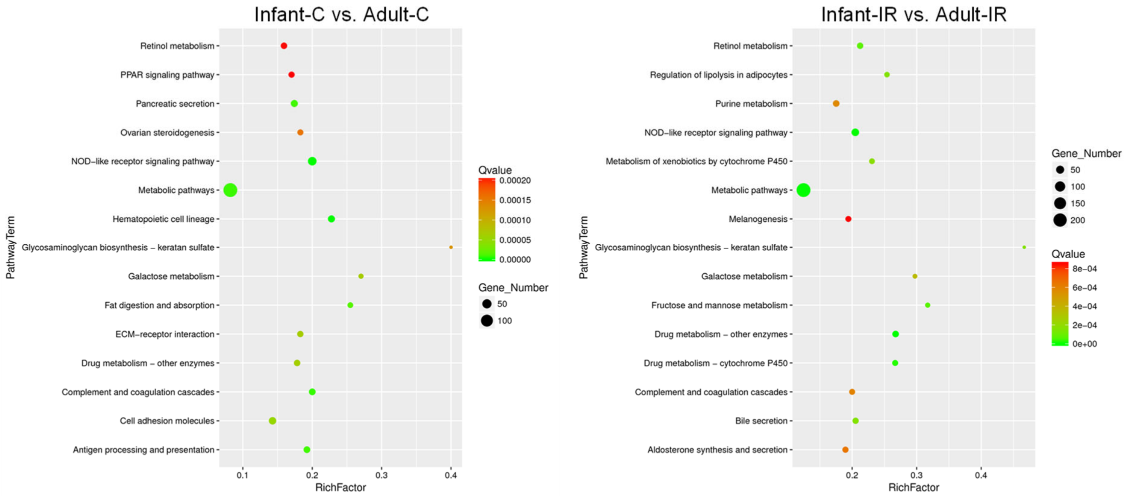

Supplementary Figure S6. Bubble charts of the KEGG classifications of assembled DEGs. Top 15 enriched KEGG pathway analysis on DEGs. Infant-C vs. Adult-C (left) and Infant-IR vs. Adult-IR (right). The number of DEGs enriched in the pathway was indicated by circle size. The Rich factor is the ratio of the number of DEGs annotated in a pathway to the number of all genes annotated in this pathway. The color saturation from green to red indicates the Q value.

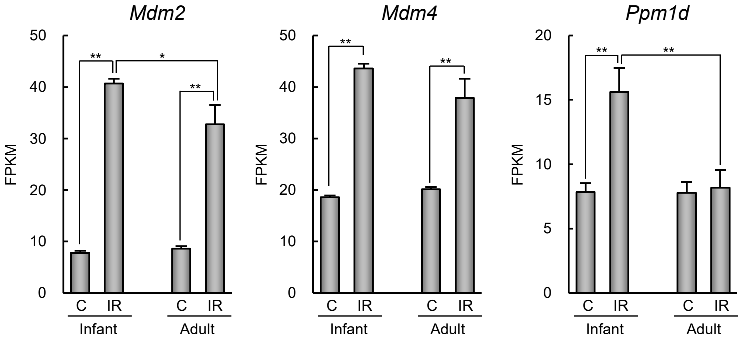

Supplementary Figure S7. FPKM values of *Mdm2*, *Mdm4*, *Ppm1d* in infant and adult stem cells after radiation exposure. Data are presented as means ± *SD* from three samples. One-way ANOVA was used to evaluate differences in means among groups. *p<0.05, **p<0.01.
